# Social and Poor-Law Problems

**Published:** 1907-11-16

**Authors:** 


### SOCIAL AND POOR-LAW PROBLEMS.

### GUARDIANS AND LOCAL CONTRACTS.

At the North-Western Poor-law Conference, recently held at Liverpool, there was a discussion, very comprehensible in the light of recent revelations, of the duty of guardians in the giving of contracts. A very instructive paper on the subject was read by Mr. A. J. Jolley, of Warrington. While the general rules for the giving of contracts and accepting of tenders are settled by the Local Government Board, the orders under which the guardians act are of considerable flexibility. Such variety is necessary to meet the differing conditions of different localities. But there is always a danger of the freedom thus allowed being abused. There is always the danger of corruption—gifts or promises from competing firms, and, as we have lately seen, sometimes demanded by guardians as a condition of their support. But this is a danger inherent in the wickedness of human nature, and no form of contract could be devised to meet it. Only a high standard of honour among guardians themselves can correct this. But there are faults into which well-meaning but ignorant guardians may fall, which Mr. Jolley pointed out. Among these the chief is the desire to spend within the union the money raised by the union. There is a quite comprehensible preference felt for giving a contract to a local firm rather than to an outsider. And, other things being equal, there is no objection to this. But, as often as not, other things are not equal. The local tenderer is not always the lowest, and while it is a wise proviso to say that the lowest tender will not inevitably be accepted, for a low price may mean an inferior quality, the assumption is that between two articles of equal merit the cheapest will be accepted. Unfortunately the proviso as to not accepting the lowest tender is often twisted to give a preference to a local firm, and even honourable men will let themselves be led into supporting such a course of action by the plea that the local firm employs local industry, and both directly and indirectly is the means of circulating money in the neighbourhood. But this principle, once accepted, tends to limit the scope of choice. Wholesome competition dies away when once it is known that outsiders have but little chance in competing for a contract. And with a practical monopoly there is the temptation of the local firm to lower the quality of the goods supplied until they in no way meet the conditions of the tender.

Another source of danger is the opening of tenders in the clerk's office as they are received. It is usually impossible to say how information as to the prices asked in the early tenders gets disseminated, but such information does often leak out, and local tenderers, by postponing the sending in of their offers, can occasionally find out what reductions of price are necessary to secure the contract. To avoid this danger it is desirable that all tenders should be sealed, and that none should be opened before the meeting at which the contract is to be decided. This is certainly a slow and often tiresome business, but it is better than having the ratepayers' money wasted and a subtle dishonesty generated through the district.

